# Relationships between body condition score, body weight and body measurements in alpacas

**DOI:** 10.1186/s13620-024-00274-z

**Published:** 2024-05-30

**Authors:** Johannes Buchallik-Schregel, Frederik Kiene, Juliane Buchallik, Hannah Marahrens, Nina Ossowski, Carolin Viktoria Schumacher, Berit Gerstel, Ulla Reimers, Martin Ganter, Matthias Gerhard Wagener

**Affiliations:** Clinic for Swine and Small Ruminants, Forensic Medicine and Ambulatory Service, University of Veterinary Medicine Hannover, Foundation, Hannover, Germany

## Abstract

**Background:**

The nutritional status in alpacas is often masked by their dense fibre coat. Its assessment is commonly approached by different body condition scores (BCS) that rely on manual palpation of defined anatomical regions. However, BCS is an important diagnostic tool to aid recognition of diseased South American camelids (SACs) and low BCS has been associated with conditions like anaemia and neutrophilia. For dose-dependent veterinary treatment, body weight (BW), that should be as accurate as possible, is required. As on-site weighing with scales is often not possible, BW can mostly only be roughly estimated. To date, it remains unclear whether BCS in alpacas provides reliable information on BW or the ratios of BW to body length commonly known as Body Mass Index (BMI) or Ponderal Index (PI). Equations to estimate BW based on body measurements are available in the literature. Nonetheless, respective equations were developed in growing alpacas or adult llamas and BCS was not included.

**Results:**

To compare six different BCS approaches and to examine the relationship between BCS and BW, body measurements and BCS scores were recorded in a herd of 105 alpacas. The examined BCS approaches showed significant (*p < 0.05*) but poor to moderate positive correlations to BW, BMI or PI. A solely visual inspection of BCS, in contrast, was not correlated with BW, BMI or PI. Equations previously developed in other studies provided an accurate estimation of BW. Multiple linear regression showed that the accuracy in predicting BW could be further increased by adding BCS data and sex.

**Conclusion:**

Our observations indicate that most selected BCS approaches are not only important measures of nutritional status but can also be used to create more accurate models for BW calculation in alpacas. The study also supports the claim that a purely visual inspection of alpacas is not an adequate method to evaluate the nutritional status of these animals.

**Supplementary Information:**

The online version contains supplementary material available at 10.1186/s13620-024-00274-z.

## Background

In recent years, the number of farms keeping South American camelids (SACs) has steadily increased in Germany [1]. A survey published by Neubert et al. in 2021 elucidated that 70% of all farms housed less than 16 animals per farm. Body weight (BW) is mostly estimated by owners and veterinarians, as on most of these small farms for SACs, scales are not accessible [1]. However, for precise dosing of medication, an accurate BW is necessary. Especially for sedation or general anaesthesia, the animal’s BW should be as accurate as possible to avoid over- or underdosing. Another example is the administration of anthelmintics, which plays an integral role in husbandry and veterinary care of SACs [2, 3]. *Levamisole*, a commonly used anthelmintic in SACs, has a small dose range (8–12 mg/kg per os), and overdosing (> 15 mg/kg) may lead to severe neurological dysfunction [2, 4].

In several species, including horses [5–7], donkeys [8], cows [9, 10] and SACs [11–14], attempts have been made to estimate BW based on body measurements. Several studies investigated the BW measurement of growing SACs, and equations with high predictive values (coefficient of determination (R²) ranging from 94.8 − 98.0%) for estimating BW have been published for a population of growing SACs [11–14]. The relationship between BW and body measurements such as thoracic circumference (TC) was also investigated in a heterogeneous population of llamas (*Lama glama)*, and equations based on TC can be used to accurately estimate BW [15]. In a study recently published by Ablondi and colleagues, the relationship between BW and body measurement was investigated in a population of growing and adult alpacas and an equation for predicting BW was published [16]. However, the resulting equation was based on a population which consisted mainly of growing animals (17 grown, 32 growing alpacas), and the nutritional status of the alpacas was not evaluated [16]. Although body condition scores (BCS) are a well established method to rate the nutritional status of SACs [17–20], these scores have not been included in equations for estimating BW. In other species such as horses [21], sheep [22] and dogs [23], correlations between BCS and BW as well as BMI have been documented. The relationship between BCS and haematological findings like anaemia or leucocytosis has been previously described in SACs, which underlines that BCS assessment is an important tool for herd management [24]. Low BCS in alpacas have also been associated with chronic disease such as tuberculosis [25, 26]. In another previous study we showed that assessing BCS based on palpation of the lumbar spine is a reproducible measurement [17]. Nonetheless, in other previously published studies investigating BCS, it was not proven that BCS correlated with BW, and there was no investigation of the association between BW and body length, Body Mass Index (BMI) or Ponderal Index (PI) [27], which are explained in the Material and Methods section in detail.

These aforementioned measures are commonly used in other species, including pigs [27], sheep [28, 29], cattle [30, 31] and camels [32] and are applied to categorise animals according to their height/length relative to their BW. To assess whether the use of these parameters could be useful for alpacas (*Vicugna pacos*), the main purpose of this study was to answer the three following questions for the species:

1. Does the BCS provide any information on BW, BMI or PI?
2. How reliable would previously published equations predict BW of alpacas in our studied population?
3. Do the factors sex, age or BCS improve the accuracy of equations predicting BW, and are these variables useful to predict the BW of alpacas under field conditions?

## Materials and methods

### Herd

The study was conducted on an alpaca farm housing more than 250 alpacas of different ages in northwestern Germany. A subset of 105 individuals was investigated for this study. The study population consisted of 60 males and 45 females. Seventeen of the 45 females were pregnant; however, further information regarding pregnancy was not available. The age of the animals ranged from 2 to 18 years, while the median age was 5 years. The examination took place in February 2023; at that time the animals were not shorn.

### Zootechnical measurements

Body weight was assessed in a subset of 105 alpacas using portable scales (Meier-Brakenberg GmbH, Extertal, Germany) and was recorded in kg with one position after the decimal point. In addition, zootechnical measurements were recorded using a metre measuring tape and a measuring stick. The measurements were taken to the nearest centimetre and were conducted for the entire population by the same two examiners.

**Fig. 1:**
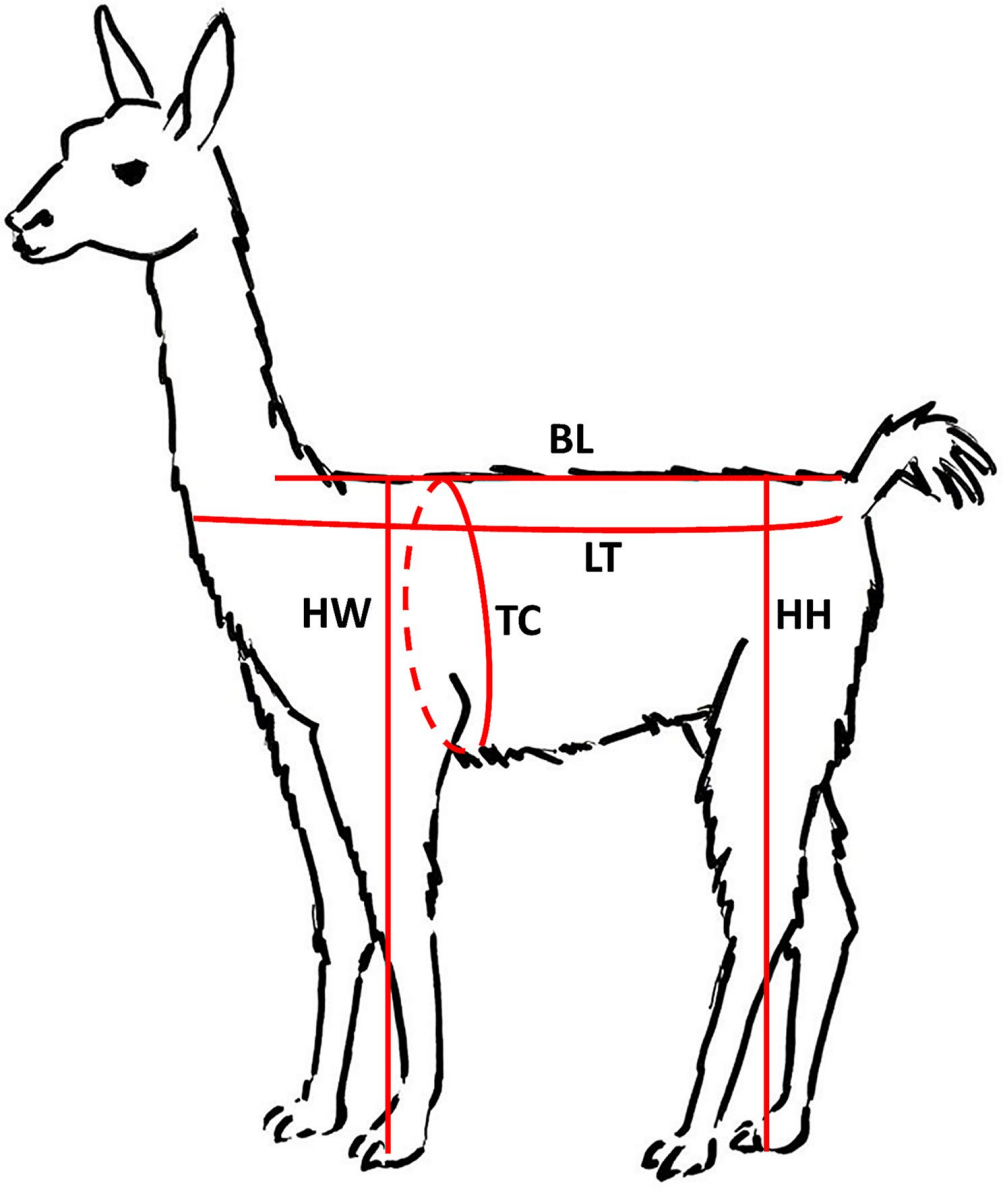
Schematic visualisation of zootechnical measurements. *Abbreviations* BL = Back length, HH = Height at hips, HW = Height at withers, LT = Length of the trunk, TC = Thoracic circumference. Graphic: L. Grimm and J. Buchallik-Schregel. For details of the individual parameters, see Table 1

The following parameters were measured based on the study of Grund et al. [11]. Respective body measurements are summed up in Table 1 and visualised in Fig. 1.

**Table 1:** Summary of zootechnical measurements according to Grund et al.[10]

Measurement	Abbreviation	Description
Thoracic circumference	TC	Circumference of the thorax, caudal of the forelimb
Length of the back	LB	Distance between the first palpable spinous process of the first thoracic vertebra to the first movable vertebra of the caudal vertebra
Length of the trunk	LT	Distance between the ventral lamina of the sixth cervical vertebra to the ischial tuber of the *Os ischii*
Height at the wither	HW	Distance between the highest palpable spinous process of the thoracic vertebrae to the ground
Height at the hip	HH	Distance between the Tuber sacrale of the *Os ilium* vertical to the ground

### Body condition scores

Body condition scores (BCS) were evaluated by seven examiners on every individual animal of the study population. The examiners had different levels of experience concerning the assessment of the BCS on SACs, but had previously been briefed on the assessment of the individual body sites. To minimise the risk of bias during the BCS evaluation, the examiners were not allowed to communicate with each other. For all used approaches, the BCS ranged between 1 (emaciated) and 5 (obese), as previously described [17]. A description of the different BCS is summarised in Table 2. The BCS was assessed based on the palpation of the five different anatomical landmarks. A visual body condition score (BCSO = body condition score optic) was estimated before touching the animals during subjective examinations performed by each examiner. A total body condition score (BCSA = body condition score all) was estimated based on the previously evaluated subjective body condition scores.

**Table 2:** Summary of body condition scores

Body condition score	Anatomical location	Description of scoring
BCSL	First lumbar vertebrae	Scored according to tissue coverage between spinous and transverse processes. With an optimal score of 3, straight connecting line between the tips of the spinous and transverse processes; if the line was concave, the given score was < 3; if it was convex, the given score was > 3.
BCSW	Withers	Palpation at withers; interpretation as with BCSL.
BCST	Caudally of the right elbow, on the less hairy area medial to the elbow	Costal bones were palpated with a flat hand. A score of 3 was defined as palpation of the costal bones, applying only slight pressure. If costal bones were palpable without applying further pressure, a score of < 3 was recorded, whereas scores > 3 were documented if further pressure was needed to palpate the costal bones.
BCSP	Pectoral muscles	Graded according to fat or muscle deposition with 1 (lean) to 5 (obese) with 3 (optimal).
BCSH	Tuber coxae	If tuber coxae were easily palpated with slight pressure, a score of 3 was recorded. If tuber coxae were palpable without applying force, a score of < 3 was recorded. If more pressure was required to palpate tuber coxae, a BCS of > 3 was documented.

*Abbreviations* BCSH = body condition score hips, BCSL = body condition score lumbar spine, BCSP = body condition score pectoral muscles, BCST = body condition score ribs, BCSW = body condition score at withers

### Statistics

All calculations were performed using R (Version 4.3.0) [33]. Continuous data were tested for normal distribution using the Shapiro-Wilk Normality Test [34].

As all different BCS locations were assessed by seven examiners, the median value of their assessed scores was calculated for each BCS for further analysis. The parameters BMI and PI were calculated by using body length (BL) and BW based on the formulas used by Hales et al. [27].

$$BMI=\frac{BW}{{BL}^{2}}$$

$$PI=\frac{BW}{{BL}^{3}}$$

Median BCS values, BMI, PI as well as BW were used to create a correlation matrix. Spearman’s correlation was applied, as the median of BCS was a non-continuous measurement. In the following, Spearman’s rho rank correlation was calculated by applying the rcorr procedure of the Hmisc-package [35]. The computed *p*-values were corrected for multiple testing using Bonferroni correction. The correlation coefficients were interpreted based on the suggestions of Akoglu [36].

To answer the question whether young (y) (2–7 years) or old (o) (10–18 years) alpacas differ in BCS, BW, BMI and PI, and whether the female (f) or male (m) sex influences these parameters, the studied population was subdivided. The combination of these two factors resulted in four groups (fo = female old, fy = female young, mo = male old, my = male young). As non-continuous data were analysed and data were not normally distributed and/or variance was inhomogeneous between groups, nonparametric tests (Kruskal-Wallis test and Dunn’s test [37]) were performed. The results of the Dunn’s test were adjusted for multiple testing using Bonferroni correction.

To answer the second question, whether previously created formulas deliver accurate estimates for bodyweight in our study population, the equations of Wurzinger et al., published in 2005, Leon et al., published in 1989, Smith et al., published in 1992, Riek et al., published in 2007 and Grund et al., published in 2018 [11–15] were used to calculate BW based on the available body measurements. The *lm* function was used to perform Linear Model Fitting. A global linear model with the following variables (TC, TC², TC³, LT, LT², LB, LB², BCST, BCSL, BCSH and BCSW) was created to predict BW. The parameters HW and HH were not used for predictive formulas, as values did not meet the assumptions of a linear model. The *dredge* function of the MuMin package was used to select potential models for estimating BW [38]. Only models with variables that had significant coefficients (*p* < 0.05) were used for further investigations. The variance inflation factor (VIF) was calculated, applying the “car-package” [39] in R for all presented models to determine whether multicollinearity was present in respective multivariable linear models. If multicollinearity was detected (VIF > 10), the respective variable was removed from the model [40]. Also the Breusch-Pagan test was performed to discover potential heteroskedasticity [41]. If heteroskedasticity was present, corresponding Wald confidence intervals were calculated using the *coeftest* function in R [42]. The adjusted R square and the residual standard error were calculated to assess the goodness of fit. For all formulas the Akaike information criterion was applied to measure the fit of the different models [43] and to select the best model with a single independent variable, with two independent variables, three independent variables and four independent variables. The results of correlation analysis were interpreted as described [36]. In the following step, Lin’s concordance correlation coefficient was calculated to assess the agreement between “measured” and “predicted” BW of the different methods [44].

## Results

### Descriptive data

Descriptive data for all animals are presented in the following table (Table 3).

**Table 3:** Descriptive data on the investigated alpaca population

	Min	Max	Median	Mean	Std. Deviation	Standard error of the median	Standard error of the mean
Age (day)	477	6656	1696	2125	1395	20.7	136
Age (year)	1.31	18.2	4.64	5.81	3.82	1.08	0.371
BW (kg)	33.6	89.0	60.4	60.8	11.8	1.90	1.15
HW (cm)	65	93.0	71	76.2	9.13	1.67	0.891
HH (cm)	67	95.0	72	77.4	9.38	1.70	0.915
TC (cm)	75	109	93	92.9	7.37	1.50	0.720
LB (cm)	61	90.0	76	75.9	5.57	1.31	0.544
LT (cm)	70	110	91	91.4	8.84	1.65	0.862
BCSA	1.5	4	3	2.86	0.440	0.367	0.043
BCSP	1.5	3.5	3	2.78	0.404	0.352	0.039
BCSH	1.5	3	2.5	2.34	0.333	0.320	0.033
BCSL	1.5	4	3	3.05	0.494	0.389	0.048
BCSO	2	4	3	2.86	0.446	0.370	0.043
BCST	1.5	4	3	2.75	0.396	0.349	0.039
BCSW	1.5	3.5	2.5	2.64	0.375	0.339	0.037

*Abbreviations* BCSA = body condition score all, BCSH = body condition score hips, BCSL = body condition score t lumbar spine, BCSO = body condition score optic, BCSP = body condition score pectoral muscles, BCST = body condition score ribs, BCSW = body condition score at withers, BW = body weight, HH = Height at the hip, HW = Height at the withers, LB = Length of the back, LT = Length of the trunk, TC = Thoracic circumference

#### Correlation between different BCSs and BW, PI and BMI

**Fig. 2:**
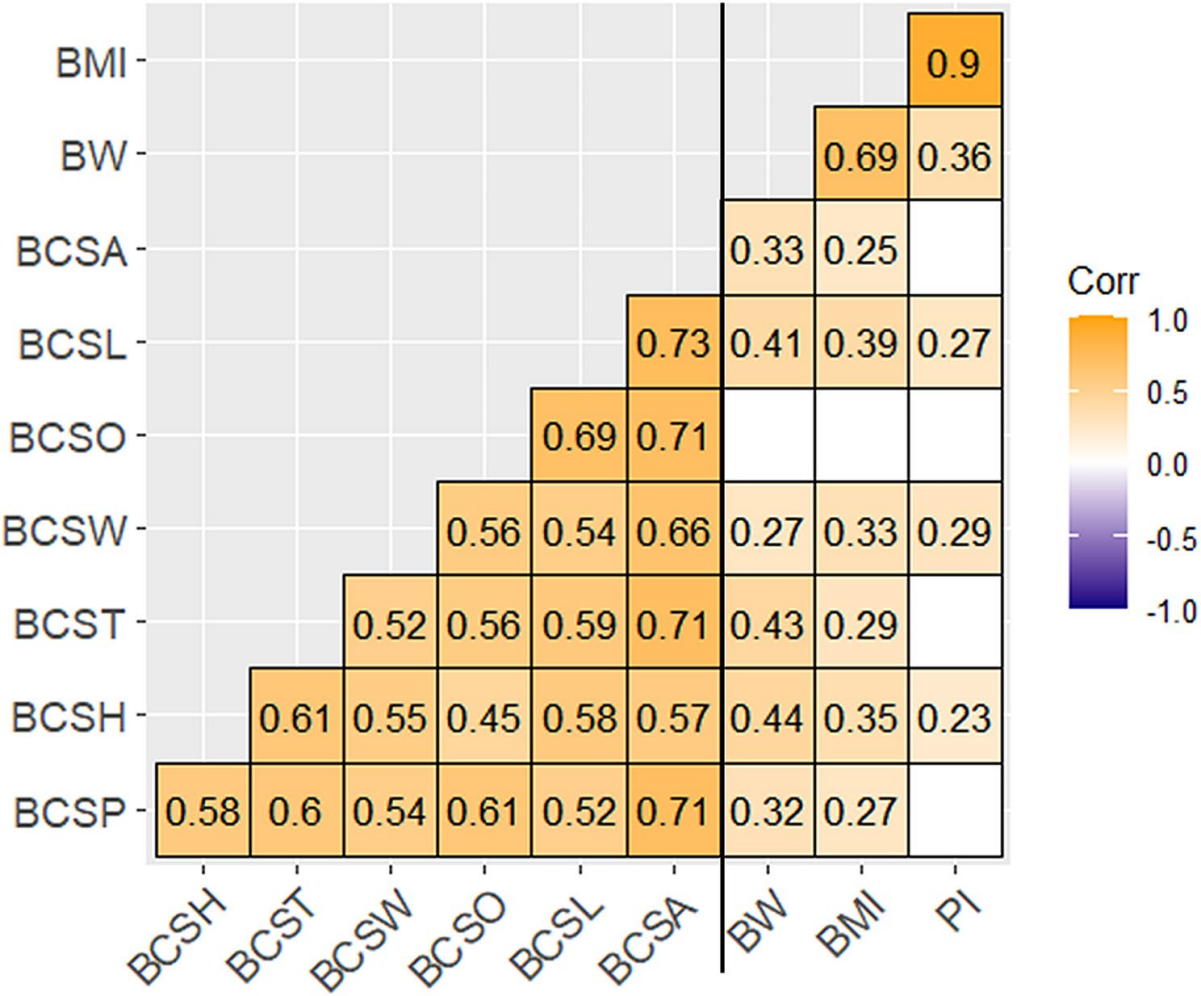
Heatmap presenting rank correlation between body condition scores, body weight and Body Mass Index and Ponderal Index. Blank fields symbolise an insignificant (*p* > 0.05) correlation between respective parameters; *n* = 105. *Abbreviations* BCSA = body condition score all, BCSH = body condition score hips, BCSL = body condition score lumbar spine, BCSO = body condition score optic, BCSP = body condition score pectoral muscles, BCST = body condition score thorax, BCSW = body condition score at withers, BW = body weight, BMI = Body Mass Index, PI = Ponderal Index

The results of the Spearman’s rank correlation are visualised in a heatmap (Fig. 2). Only correlations with *P* < 0.05 are shown. Otherwise, respective fields of the heatmap are left blank. Correlation analysis showed that BCSA was positively correlated with BW (r² = 0.33, *p <* 0.001) and BMI (r² = 0.25, *p =* 0.009). Additionally, BCSL was positively correlated with BW (r² = 0.41, *p <* 0.001), BMI (r² = 0.39, *p <* 0.001) and PI (r² = 0.27, *p =* 0.005). The parameter BCSW was positively correlated with BW (r² = 0.27, *p =* 0.005), BMI (r² = 0.33, *p <* 0.001) and PI (r² = 0.29, *p =* 0.003). Positive correlations were detected between BCST and BW (r² = 0.43, *p <* 0.001) as well as BCST and BMI (r² = 0.29, *p =* 0.003), while positive correlations were identified between BCSH and BW (r² = 0.44, *p <* 0.001), BMI (r² = 0.35, *p <* 0.001) and PI (r² = 0.23, *p =* 0.002). Furthermore, the parameter BCSP was correlated with BW (r² = 0.32, *p <* 0.001) and BMI (r² = 0.35, *p =* 0.004). There was no significant correlation found between BCSO and BW, BMI and PI.

#### Influence of age and sex on different body condition scores

**Fig. 3:**
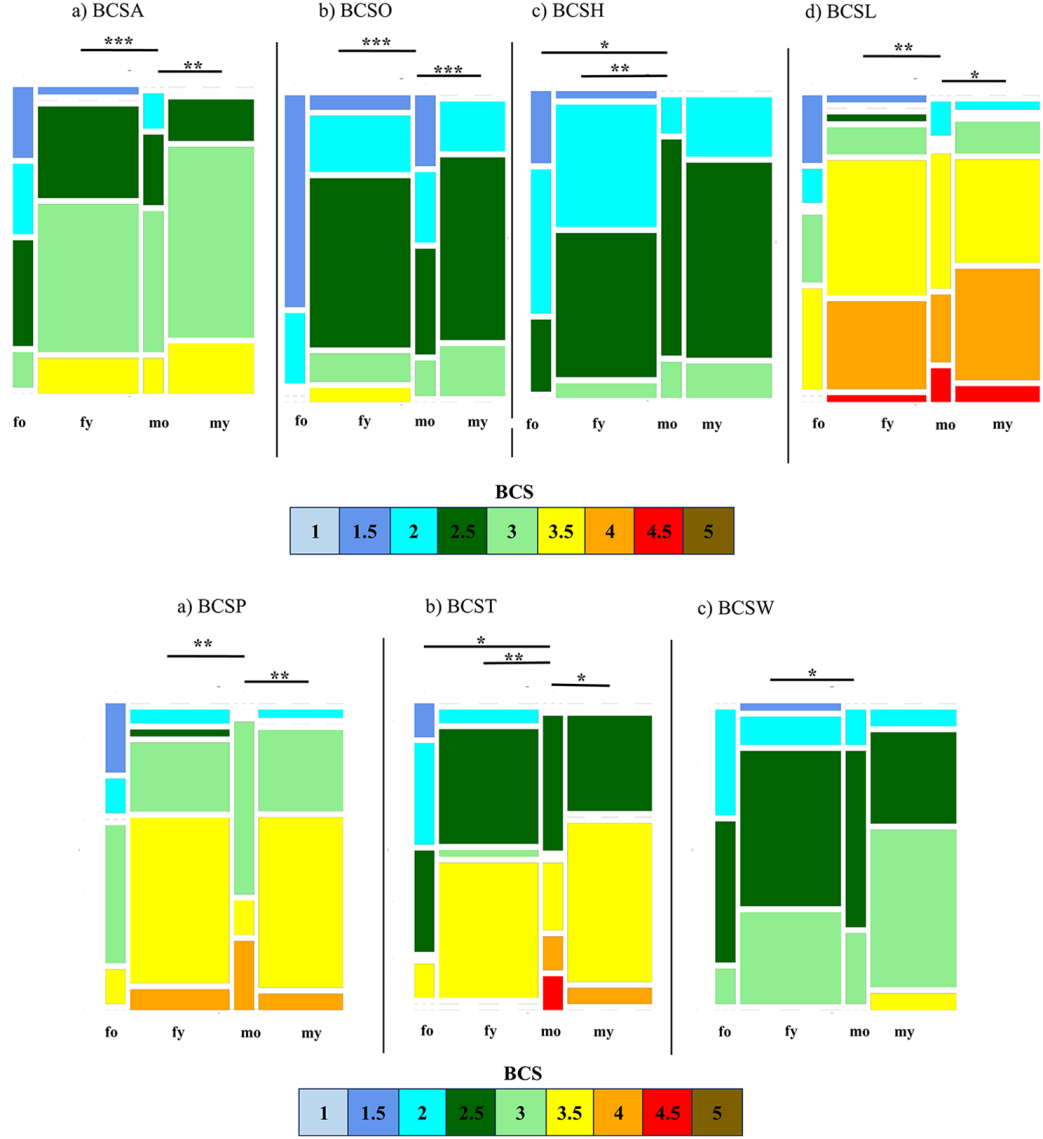
Distribution of BCS in female and male alpacas originating from different age groups. Group size: fo (females old) *n =* 8, fy (females young) *n =* 34, mo (males old) *n =* 8, my (males young) *n =* 40;* indicates significantly different median values between groups (*p* < 0.05);** indicates significantly different median values between groups (*p* < 0.01);*** indicates significantly different median values between groups (*p* < 0.001). *Abbreviations*: BCSA = body condition score all, BCSO = body condition score optic, BCSH = body condition score hips, BCSL = body condition score at lumbal spine. The width of the columns represents the number of examined animals

The composition of groups was visualised using a mosaic plot (Fig. 3a and b). The comparisons for all four age- and sex-related groups by Kruskal-Wallis tests were significant for all six BCS approaches (*p* < 0.05). In the following, Dunn’s tests were applied to identify differences between the single groups. The median BCSP was lower (*p* < 0.01) in mo (median = 2.5, SD = 0.23, *n* = 8) compared to my (median = 3, SD = 0.42, *n* = 40) and fy (median = 3, SD = 0.3, *n* = 34). The median BCST was smaller (*p* < 0.05) in mo (median = 2.25, SD = 0.46, *n* = 8) than in my (median = 2.88, SD = 0.3, *n* = 40), fy (median = 3, SD = 0.29, *n* = 34) and fo (median = 2.75, SD = 0.56, *n* = 8). Furthermore, the median BCSW was lower (*p* < 0.05) in mo (median = 2.5, SD = 0.35, *n* = 8) than in fy (median = 3, SD = 0.35, *n* = 34). The median BCSA was smaller (*p* < 0.05) in mo (median = 2, SD = 0.38, *n* = 8) compared to fo (median = 2.5, SD = 0.27, *n* = 8) and fy (median = 2.5, SD = 0.29, *n* = 34). The BCSO was lower (*p* < 0.001) in mo (median = 2, SD = 0.23, *n* = 8) than in my (median = 3, SD = 0.42, *n* = 40) and fy (median = 2.5, SD = 0.29, *n* = 34). Furthermore, the median BCSH was smaller in mo (*p* < 0.01) (median = 2, SD = 0.38, *n* = 8) than in fy (median = 2.5, SD = 0.29, *n* = 34) and fo (median = 2.5, SD = 0.27, *n* = 8). Also, the median BCSL was lower (*p* < 0.05) in mo (median = 2.5, SD = 0.64, *n* = 8) than in my (median = 3, SD = 0.45, *n* = 40) and fy (*p* < 0.01) (median = 3, SD = 0.44, *n* = 34). Differences regarding BW, BMI and PI were not detected between the groups (*p* > 0.05) (see Supplementary Fig. 1).

#### Formulas to predict body weight based on body measurements

**Fig. 4:**
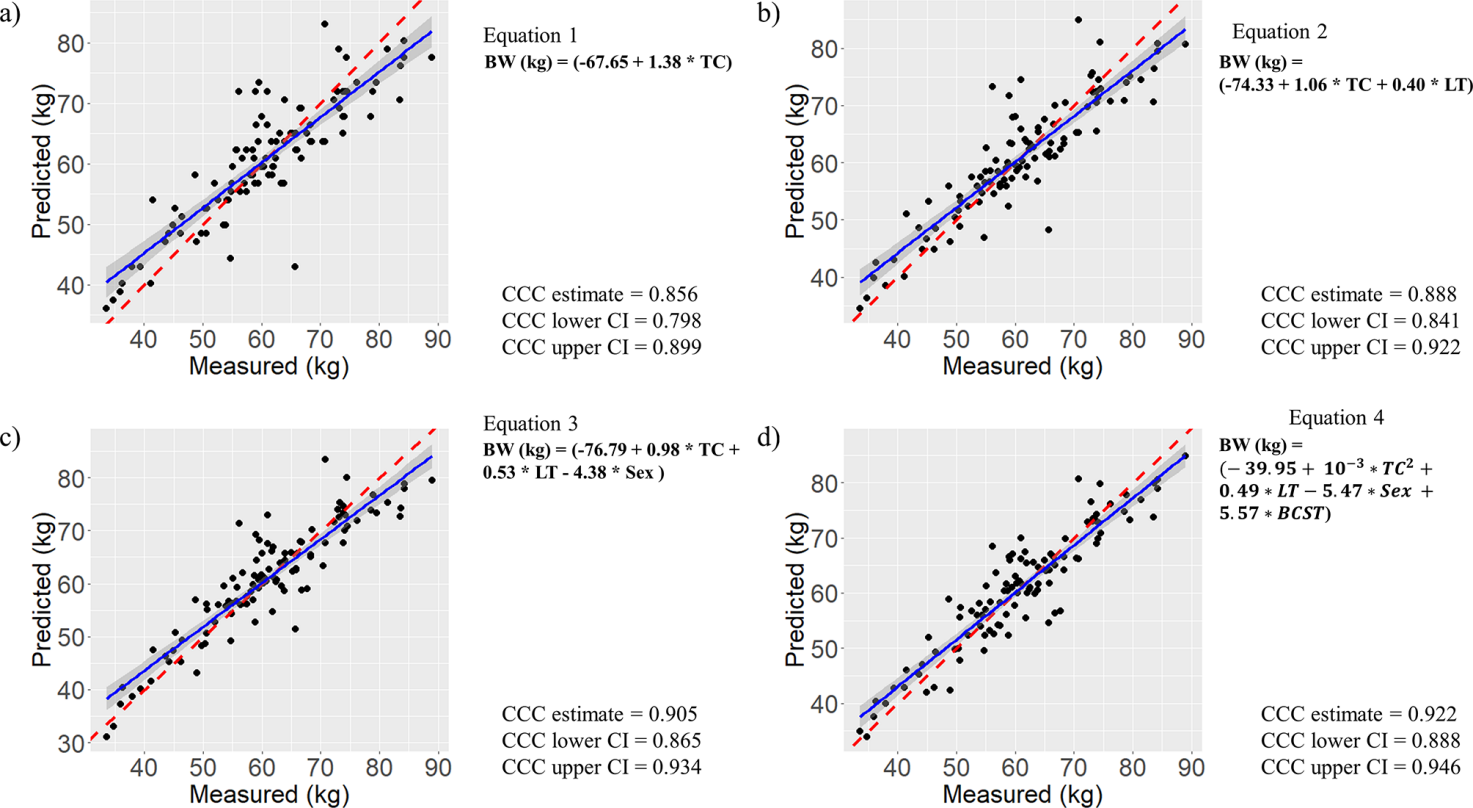
Visualisation of Lin´s concordance correlation coefficient of four equations containing up to four independent variables. **a**) Best prediction of BW based on a single independent variable, **b**) best prediction of BW based on two independent variables, **c**) best prediction of BW based on three independent variables, **d**) best prediction of BW based on four independent variables; for all equations *n* = 105. Abbreviations: BW = Body weight, BCST = Body condition score thorax, TC = Thorax circumference (cm), CCC = Concordance correlation coefficient, CI = Confidence interval, LT = Length trunk (cm), Sex: male = 0, female = 1

Applying the previous published equation showed that these formulas moderately predicted BW in the investigated population (Table 4). However, BW was estimated by our own developed equations more accurately. Adding the independent variables “sex” and “BCST” improved the accuracy of the equations. The most accurate equations, based on the numbers of independent variables, are shown. Further equations are accessible as supplementary material (Supplementary Table 1). Results of Lin´s concordance correlation coefficient were visualised in Fig. 4. Based on the results of Lin´s concordance correlation coefficient, only two equations (Eq. 3 and Eq. 4) delivered predictions with moderate precision and accuracy. The other formulas delivered only poor prediction.

**Table 4:** Formulas predicting body weight of alpacas based on different body measurements

Equation	adjusted *R*²	SE	AIC	CCC estimate	95% CI
Published equations for BW calculation^1^					
(Grund ) BW (kg) $=$ ($3.33+8.34*{10}^{-5}*{TC}^{3}$)	0.74	6.09	681.2	0.636	0.555–0.718
(Wurzinger) BW (kg) $=(-26.31+{10}^{-3}*7.38*{TC}^{2}+\left(-0.63*TC\right)+\left(-4.26*{10}^{-3}*{LT}^{2}\right)+1.34*LT)$	0.78	5.58	663.0	0.778	0.706–0.834
(Wurzinger 2) BW (kg) $=$ ($30.94+\left({10}^{-3}*{3.88*TC}^{2}\right)+\left(0.49*TC\right))$	0.74	5.98	667.6	0.483	0.397–0.561
$\left(\text{L}\text{e}\text{o}\text{n}\right) \text{B}\text{W} \left(\text{k}\text{g}\right) = \left(8.7*{10}^{-4}*{TC}^{2.46}\right)$	0.74	6.04	679.5	0.861	0.803–0.904
$\left(\text{R}\text{i}\text{e}\text{k}\right) \text{B}\text{W} \left(\text{k}\text{g}\right) = (12.61+\left(-0.82*TC\right)+\left(0.02*{TC}^{2}\right))$	0.74	6.02	679.0	0.142	0.105–0.179
(Riek 2) BW (kg) $$=$$$$(78.79+\left(-2.82*HW\right)+\left(0.03*{HW}^{2}\right))$$	0.70	6.47	694.0	0.477	0.388–0.557
$\left(\text{S}\text{m}\text{i}\text{t}\text{h}\right) \text{B}\text{W} \left(\text{k}\text{g}\right) = \left(1.005*{10}^{-3}*{TC}^{2.424}\right)$	0.74	6.03	679.4	0.859	0.799–0.901
$\left(\text{S}\text{m}\text{i}\text{t}\text{h}2\right) \text{B}\text{W} \left(\text{k}\text{g}\right) = \left(1.23*{10}^{-3}*{TC}^{2.385}\right)$	0.74	6.03	679.2	0.860	0.801–0.903
New equations					
(Eq. 1) BW (kg) = $(-67.65+1.38*TC)$	0.75	5.96	676.9	0.856	0.798–0.899
(Eq. 2) BW (kg) = $(-74.33+1.06*TC+0.40*LT)$	0.79	5.37	655.8	0.888	0.841–0.922
(Eq. 3) BW (kg) = $\left(-76.79+0.98*TC+0.53*LT-4.38*Sex\right)$	0.82	4.99	641.8	0.905	0.865–0.934
(Eq. 4) BW (kg) = $(-39.95+{10}^{-3}*{TC}^{2}+0.49*LT-5.47*Sex+5.57*BCST)$	0.85	4.59	624.0	0.922	0.888–0.946

*Abbreviations* AIC = Akaike information criterion, BW = Body weight, BCST = Body condition score thorax, CCC = Concordance correlation coefficient, CI = Confidence interval, HW = Height at withers (cm), LT = Length trunk (cm), SE = Standard Error of the Mean, Sex = Sex of Alpaca (male = 0, female = 1), TC = Thorax circumference (cm)

^1^Published equations refer to the following publications: [11–15, 11–13, 15, 29].

## Discussion

A low nutritional status is a common problem in SACs and can be attributed to chronic infectious diseases, endoparasites, dental problems or poor feeding management [45]. A retrospective study of necropsy data from SACs presented to our clinic over a 16-year period showed that more than half of the animals were thin or cachectic, which often went undetected for a long time [46]. Therefore, regular examination of the BCS by the owner is recommended to detect emaciated alpacas and llamas in time.

In another previous study we found that pathological conditions like anaemia and leucocytosis were more frequently detected in alpacas with low BCS [24]. Body condition scores assessed on the loin are a reproducible measure, do not require further equipment and can be performed not only by veterinary educated personnel but also by well instructed animal owners to rate the nutritional status of alpacas [17]. However, BCS measurements are not commonly performed by the owners of alpacas [1].

Different locations have been described to evaluate the BCS in alpacas [45], including the lumbar spine [17], the thorax, the chest, the hips and inner thigh [20]. A moderate positive correlation between BCSL and BW was detected in a population of alpacas assigned for treatment at a veterinary hospital [24].

To our knowledge, this is the first study investigating the interaction of different BCS with BW, BMI and PI. Body Mass Index and PI are calculated in other species based on crown-rump length [27, 30], which describes the complete length of the spine. For the calculation of BMI and PI in alpacas it is preferable to use the length of the back, which is the distance between the first palpable spinous process of the first thoracic vertebra and the first movable vertebra of the caudal vertebra. This is because alpacas have long and often thin necks and their spines are L-shaped. This complicates the accurate measurement of this parameter.

The results of our study indicated that BCSL, BCST and BCSH correlated moderately positively with BW. In contrast, BCSO was neither significantly correlated with BW, nor with BMI nor with PI. These results support our claim that nutritional status of alpacas can only be determined reliably if defined anatomical regions of the animal are palpated on a regular basis. This claim has also been presented before and is common sense in basic alpaca farming [20, 45]. However, the parameter BW is not only influenced by the nutritional status of the animals, but also the actual filling of the forestomach, or, in pregnancy, of the uterus [47].

The investigated population had a virtually homogenous nutritional status. As the study farm is a large farm that has been in existence for many years and the livestock owners regularly undergo further training and education, it can be assumed that the management is professional. The lowest median BCS were recognised in the subgroup of old males (mo). Based on our investigations, an obvious reason for this observation was not detectable. According to McConnell and Hoffman, a lower BCS in older alpacas is a common finding [48]. The most obvious explanation for low BCS in alpacas are infections with endoparasites [49]. Endoparasite infection as a reason for low BCS seems unlikely, as this alpaca population was intensively monitored by veterinary professionals. Dental disorders are also a common finding, especially in older alpacas [50] and the negative effects of periodontal disease on BCS have already been characterised [51]. As both studies did not identify a higher risk for males of developing dental disorders, it remains speculative whether dental disorders might explain our observation. As all males were uncastrated, competition between males might be another explanatory variable for our observation. It has been documented that male alpacas fight for limited resources such as food [52]. As feeding and feed of the animals were not examined, the explanation for lower BCS in the mo-group based on higher competition for feed remains speculative. Lower BCS might also originate from trace element supply, chronic disease or frequent breeding. The sex-dependent effect of age on BCS has not been well characterised in alpacas until now. It has been described in dogs that ageing females show elevated frequencies of high BCS, but in ageing male dogs ageing was not associated with elevated frequencies of high BCS [53, 54]. However, the sex-dependent mechanism of age on BCS remains unknown. Overall, it is important not to overinterpret this result, as the mo subgroup of the study population only consisted of eight individuals.

The second question was whether the previously published equations used to predict BW based on body measurements were accurate to estimate BW in our study population (See Table 4). The results indicate that a moderate to good estimation can be performed based on all equations. Nevertheless, these were developed in growing alpacas [11, 12] or llamas (*Lama glama*) [13, 14]. In accordance with previous results, equations based on the TC served as the best predictors for BW.

As expected, the accuracy of our estimations was improved when further independent variables were added to the equation. The third research question could be confirmed, since both sex and BCS influenced the prediction of BW in alpacas. Age of the alpacas was not identified as a potential predictive variable. As in other species, males tend to have a higher BW than females [8], which is expressed in Eqs. 3 and 4. The term “-4.38*Sex” expresses that males with same TC and same TL tended to be on average 4.38 kg heavier than females. The observed sex-dependent effect of age on BCS was only observed in eight individuals, representing less than 15% of all males. It is obvious that alpacas with higher BCS are heavier than alpacas with a lower BCS and the same body size. As expressed in the equations, other measurements such as TC or TL describe the body size and BW much better. To our knowledge, the presented equations are the first equations of their kind including BCS for BW prediction. Although equations with BCST had the highest predictive outcome, other BCS such as BSCL and BSCH also improved the predictive outcome of the presented equations (Supplementary Table 1).

The most obvious method of estimating the BW of an alpaca is to estimate the BW based on visual judgement and experience, but the nutritional status of alpacas is often wrongly assessed on the basis of visual impressions alone, which cannot be standardised. Hilton et al. demonstrated that even experienced veterinarians and owners estimating body weight solely based on visual impression yielded significantly differing results [18].

It must be noted that only healthy animals on one farm were investigated. All in all, the nutritional status of the complete herd can be rated as good, and only single animals were emaciated. Therefore, it might also be relevant to investigate a larger population of alpacas with a broader range in BCS on different farms in future studies. At the same time, the prediction would have been more accurate if the sample size had been higher. Only adult alpacas were investigated in this study. Therefore, future studies also need to clarify if the developed equations can be transferred to estimate BW of growing alpacas, as the parameter BCS was not applied in previous studies [11, 12, 14]. Furthermore, it needs to be clarified if BCS is an adequate method to rate the nutritional status of growing alpacas. Additionally, only intact males and females were investigated in those studies and it is not known whether neutering may influence the accuracy of previously developed equations. The effects of neutering on BCS development or fat deposition are less well documented in SACs than in other species [53, 55–57]. This is of particular interest, as in the German population approximately one in three male alpacas has been neutered and more than 50% of all male llamas have been neutered [1].

Therefore, future studies with a larger population should be performed to verify our developed equations. For this reason, a more inhomogeneous population originating from different farms should be selected to create a more accurate equation to estimate the BW of alpacas. At the start of the practical application, it is recommended to compare the calculated weights with the weights determined using scales, regardless of the equation used. This will provide the user with the necessary certainty in the estimation. Future studies also need to confirm whether predicted BW (based on measurements) or estimated BW (based on visual impression) would be the more accurate method for estimating BW in SACs.

## Conclusion

This study showed that an evaluation of nutritional status in alpacas based solely on visual impression is not an adequate method. The better method to evaluate nutritional status in alpacas is to palpate defined anatomical locations such as the loin, the withers or the thorax. However, correlations between BCS and BW were only weak to moderate. Previously published equations estimated BW of our populations quite accurately. Thoracic circumference is the most important single variable to predict the BW of alpacas, and prediction can be improved if further variables such as LT, sex or BCST are added to the equation.

### Electronic supplementary material

Below is the link to the electronic supplementary material.

Supplementary Material 1

## Data Availability

The datasets used and/or analysed during the current study are available from the corresponding author on reasonable request.

## Abbreviations

BCSA: Body condition score all
BCSP: Body condition score pectoral muscle
BCSH: Body condition score tuber coxae
BCSL: Body condition score lumbar spine
BCSO: Body condition score optic
BCST: Body condition score thorax
BCSW: Body condition score withers
BL: Back length
BMI: Body Mass Index
BW: Body weight
CCC: Lin´s Concordance Correlation Coefficient
HH: Height at hips
HW: Height at withers
LT: Length of the trunk
PI: Ponderal Index
SAC: South American camelid
TC: Thoracic circumference
