# Supplementary material for: Relationships between body condition score, body weight and body measurements in alpacas
Source: Ir Vet J. 2024 May 30;77:11. doi: 10.1186/s13620-024-00274-z (PMC11138088; doi:10.1186/s13620-024-00274-z)
Supplement: Supplementary file 1 — Supplementary Material 1 [file 13620_2024_274_MOESM1_ESM.docx]

**Supplementary material**

Relationships between Body Condition Score, body weight and body measurements in alpacas

Authors: Johannes Buchallik-Schregel*, Frederik Kiene, Juliane Buchallik, Hanna Maraharens, Carolin Viktoria Schumacher, Berit Gerstel, Ulla Reimers, Martin Ganter, Matthias Gerhard Wagener

Email-Adress corresponding author: johannes.schregel@tiho-hannover.de

Affiliation: Clinic for Swine, Small Ruminants, Forensic Medicine and Ambulatory Service, University of Veterinary

Medicine Hannover, Foundation, Hannover, Germany

Supplementary figure 1


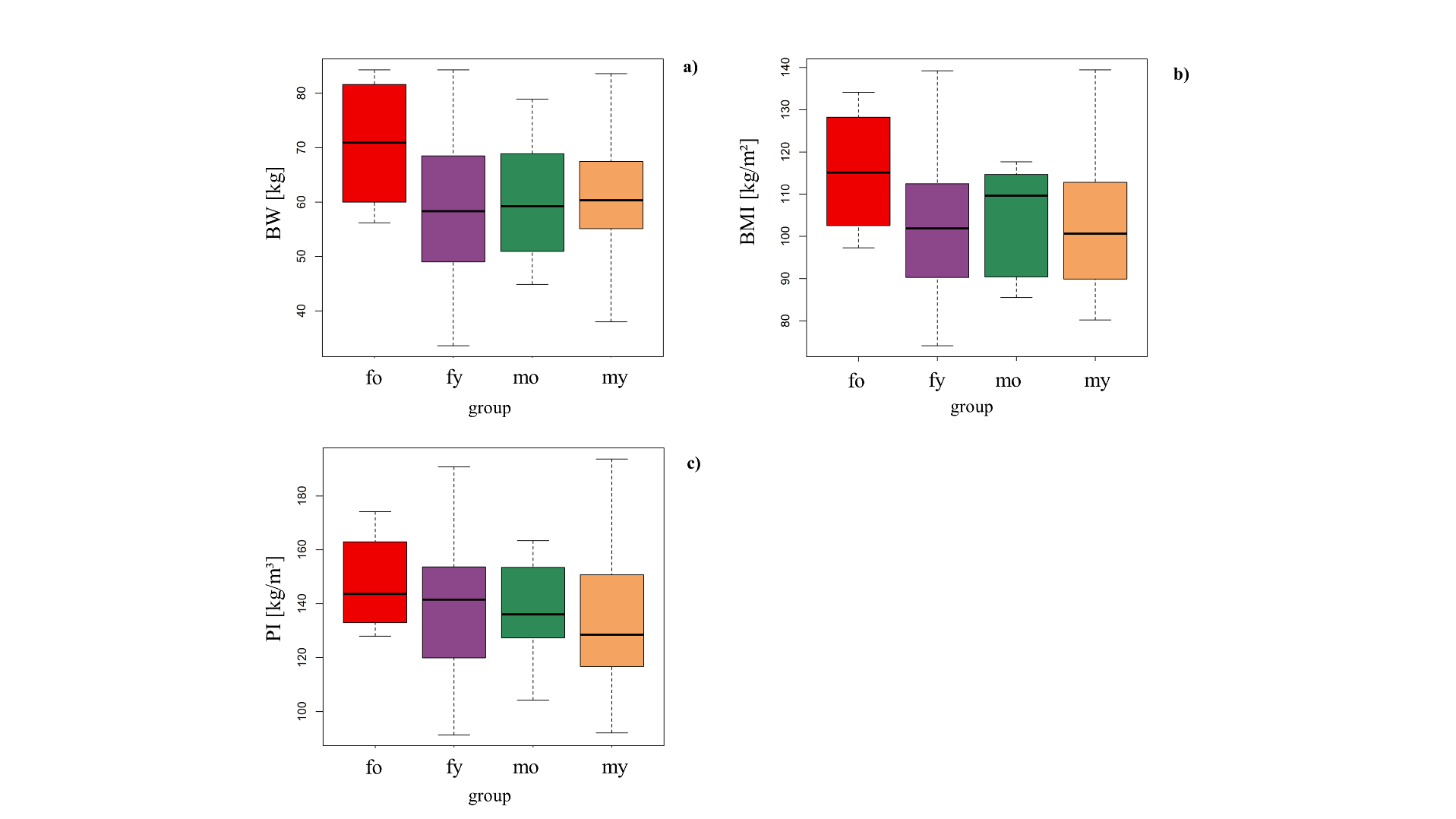


Differences between groups were not detected

Group size: fo (females old) *n =* 8, fy (females young) *n =* 34, mo (males old) *n =* 8, my (males young) *n =* 40

Abbreviations: BW = Body weight, BMI = Body-mass-index, BMI_OWN_ = Body-mass-index Own, PI = ponderal index

Supplementary table 1

| Equation | adjusted R² | Se | AIC | CCC estimate | CCC lower CI | CCC upper CI |
| --- | --- | --- | --- | --- | --- | --- |
| Literature | | | | | | |
| BW (kg) $= -39.7+{10}^{-3}*4.99*{TC}^{2}+0.5*TL+\left( -5.6*sex \right)+5.77*BCSH$ | 0.84 | 4.7 | 629.8 | 0.918 | 0.883 | 0.943 |
| BW (kg) $= -35.0+{10}^{-3}*5.20*{TC}^{2}+4.8*TL+\left( -4.89*sex \right)+2.89*BCSL$ | 0.83 | 4.84 | 635.0 | 0.913 | 0.875 | 0.939 |

Abbreviations: BW = Body weight, BCSH = Body conditioning score hip, BCSL = Body conditioning score lumbal spine, CCC = Concordance correlation coefficient, CI = Confidence interval, LT = Length trunk (cm), TC= Thorax circumference (cm)
